# A Benchmark for Data Imputation Methods

**DOI:** 10.3389/fdata.2021.693674

**Published:** 2021-07-08

**Authors:** Sebastian Jäger, Arndt Allhorn, Felix Bießmann

**Affiliations:** Beuth University of Applied Sciences, Berlin, Germany

**Keywords:** data quality, data cleaning, imputation, missing data, benchmark, MCAR, MNAR, MAR

## Abstract

With the increasing importance and complexity of data pipelines, data quality became one of the key challenges in modern software applications. The importance of data quality has been recognized beyond the field of data engineering and database management systems (DBMSs). Also, for machine learning (ML) applications, high data quality standards are crucial to ensure robust predictive performance and responsible usage of automated decision making. One of the most frequent data quality problems is missing values. Incomplete datasets can break data pipelines and can have a devastating impact on downstream ML applications when not detected. While statisticians and, more recently, ML researchers have introduced a variety of approaches to impute missing values, comprehensive benchmarks comparing classical and modern imputation approaches under fair and realistic conditions are underrepresented. Here, we aim to fill this gap. We conduct a comprehensive suite of experiments on a large number of datasets with heterogeneous data and realistic missingness conditions, comparing both novel deep learning approaches and classical ML imputation methods when either only test or train and test data are affected by missing data. Each imputation method is evaluated regarding the imputation quality and the impact imputation has on a downstream ML task. Our results provide valuable insights into the performance of a variety of imputation methods under realistic conditions. We hope that our results help researchers and engineers to guide their data preprocessing method selection for automated data quality improvement.

## 1 Introduction

In recent years, complex data pipelines have become a central component of many software systems. It has been widely recognized that monitoring and improving data quality in these modern software applications is an important challenge at the intersection of database management systems (DBMSs) and machine learning (ML) (Schelter et al., 2018a; Abedjan et al., 2018). A substantial part of the engineering efforts required for maintaining large-scale production systems is dedicated to data quality, especially when ML components are involved (Sculley et al., 2015; Böse et al., 2017).

Poor data quality can quickly break software applications and cause application downtimes, often leading to significant economic costs. Moreover, poor data quality can foster unfair automated decisions, which marginalize minorities or have other negative societal impacts (Stoyanovich et al., 2020; Yang et al., 2020; Bender et al., 2021). For this reason, many researchers started investigating to what extent monitoring of data quality can be automated (Abedjan et al., 2016; Baylor et al., 2017; Schelter et al., 2018b; Rukat et al., 2020). While some aspects of such monitoring, such as the consistency of data types, are easy to automate, others, such as semantic correctness^1^, are still the subject of active research (Biessmann et al., 2021). However, even if automatic monitoring tools, such as those proposed in the work of Schelter et al. (2017), would be used, a central challenge remains: How can we automatically fix the detected data quality issues?

One of the most frequent data quality problems is *missing values* (Kumar et al., 2017). Reasons for incomplete data are manifold: data might be accidentally not recorded, lost through application or transmission errors, intentionally not filled in by users, or result from data integration errors. Throughout the past few decades, researchers from different communities have been contributing to an increasingly large arsenal of methods to impute missing values. Statisticians laid the theoretical foundations for missing value imputation (Rubin, 1976) by describing different missingness patterns (more details are given in Section 3.2). Statistical approaches have been proposed to handle missing values (Schafer and Graham, 2002). Simple strategies include dropping incomplete observations or replacing missing values with constant mathematically valid values. While this might be a reasonable solution to ensure robust functioning of data pipelines, such approaches often reduce the amount of available data for downstream tasks and, depending on the missingness pattern, might also bias downstream applications (Stoyanovich et al., 2020; Yang et al., 2020) and, thus, further decrease data quality (Little and Rubin, 2002; Schafer and Graham, 2002).

Another line of imputation research in the statistics community focuses on multiple imputation (MI) (Rubin, 1987). In MI, one replaces missing values with multiple predictions from an imputation model. Those M>1 complete datasets can be used to assess the uncertainty of imputed values. The most popular and widely used MI technique is multiple imputation by chained equations (MICE) (Little and Rubin, 2002; van Buuren, 2018), which is very flexible and can be implemented with different models. While some applications can benefit from this uncertainty information, integrating this uncertainty information in data pipelines can be challenging. From a practitioner’s point of view, point estimates are much simpler to integrate into conventional data pipelines. This is why we restrict our analysis to point estimate imputations. Note, however, that all the experiments conducted in this work could, in principle, also be evaluated with respect to their uncertainty estimates in a MICE setting, using the examined imputation methods as the model underlying the MICE estimator.

More recently, also ML approaches have increasingly been used for imputation. Popular methods include k-nearest neighbors (*k*-NNs) (Batista and Monard, 2003), matrix factorization (Troyanskaya et al., 2001; Koren et al., 2009; Mazumder et al., 2010), random-forest–based approaches (Stekhoven and Bühlmann, 2012), discriminative deep learning methods (Biessmann et al., 2018), and generative deep learning methods (Shang et al., 2017; Yoon et al., 2018; Li et al., 2019; Nazábal et al., 2020; Qiu et al., 2020).

Most imputation studies provide solid experimental evidence that the respective proposed method in the application setting investigated outperforms other competitors’ baselines. Yet, it remains hard to assess which imputation method consistently performs best in a large spectrum of application scenarios and datasets under realistic missingness conditions. In particular, most benchmarks do not systematically report and compare both imputation quality and the impact of the imputation on downstream ML applications with baselines in a wide range of situations.

In this article, we aim at filling this gap. We benchmark a representative set of imputation methods on a large number of datasets under realistic missingness conditions with respect to imputation quality and the impact on the predictive performance of downstream ML models. For our experiments, we use 69 fully observed datasets from OpenML (Vanschoren et al., 2013) with numeric and categorical columns. Each dataset is associated with a downstream ML task (binary classification, multiclass classification, and regression). We run experiments by artificially introducing varying fractions of missing values of the three missingness patterns (MCAR, MAR, and MNAR, see also Section 3). We then measure both the imputation performance and impact on downstream performance in two application scenarios: 1) missing values in the test data; i.e., we train on complete data and corrupt (and impute) only test data and 2) both training and test data have missing values; i.e., we train and test on corrupted data.

The rest of this article is structured as follows. In Section 2, we review the related work on imputation benchmarking efforts and continue in Section 3 with an overview of the missingness conditions and imputation methods investigated in this study. A detailed description of our benchmark suite and its implementation follows in Section 4. The results of our experiments are described and visualized in Section 5. We then highlight the key findings in Section 6 and, finally, draw our conclusions in Section 7.

## 2 Related Work

The body of literature that is related to our work consists of two types of studies. Some focus on presenting new or improved imputation methods and compare them with existing and baseline approaches in broader settings, similar to benchmark papers (Bertsimas et al., 2017; Zhang et al., 2018). Others are benchmark studies and compare imputation strategies (Poulos and Valle, 2018; Jadhav et al., 2019; Woznica and Biecek, 2020). However, both have in common that they often focus on specific aspects or use cases and do not aim at an extensive comparison.

Poulos and Valle (2018) compared the downstream task performance on two binary classification datasets (N=48,842, and N=435) with imputed and incomplete data. Therefore, they varied the amount of MCAR and MNAR values from 0% to 40% in categorical features. For the imputation, they used six models: mode, random, *k*-NN, logistic regression, random forest, and SVM. The authors optimize the hyperparameters for one of the three downstream tasks but not for the imputation models. They conclude that using a *k*-NN imputation model performs best in most situations.

Similarly, Jadhav et al. (2019) compare seven imputation methods (random, median, *k*-NN, predictive mean matching, Bayesian linear regression, linear regression, and non-Bayesian) without optimizing their hyperparameters based on five small and numeric datasets (max. 1,030 observations). The authors discuss different missingness patterns but do not state which one they used in their experiments. However, they measured the methods’ imputation performance for 10% to 50% missing values. Again, the authors show that *k*-NN imputation is best independent of the dataset and missingness fraction.

Woznica and Biecek (2020) evaluate and compare seven imputation methods (random, mean, softImpute, miss-Forest, VIM kknn, VIM hotdeck, and MICE) combined with five classification models regarding their predictive performance. Therefore, they use 13 binary classification datasets with missing values in at least one column, which is why they do not know the data’s missingness pattern. The amount of missing values ranges between 1% and about 33%. In contrast to the work of Poulos and Valle (2018) and Jadhav et al. (2019), the authors could cope with the situation where only incomplete data are available for training. In their setting, they could not find a single best imputation method. However, they show that the combination of the imputation method, downstream model, and metric (F1 or AUC) influences the results.

The following two articles differ from others because they aim to compare the proposed method against the existing approaches. Zhang et al. (2018) implement an iterative expectation-maximization (EM) algorithm that learns and optimizes a latent representation of the data distribution, parameterized by a deep neural network, to perform the imputation. They use ten classification and three regression task datasets and 11 imputation baselines (zero, mean, median, MICE, miss-Forest, softImpute, *k*-NN, PCA, autoencoder, denoising autoencoder, and residual autoencoder) for comparison. The authors conducted both evaluations, imputation and downstream task performance, with 25%, 50%, and 75% MNAR missing values and showed that their method outperforms the baselines.

To the best of our knowledge, Bertsimas et al. (2017) gave the largest and most extensive comparison, although they focused on introducing an imputation algorithm and presented its improvements. The proposed algorithm cross validates the choice of the best imputation method out of *k*-NN, SVM, or tree-based imputation methods, where the hyperparameters are also cross validated. The authors then benchmarked their approach on 84 classification and regression tasks against five imputation methods: mean, predictive mean matching, Bayesian PCA, *k*-NN, and iterative *k*-NN. They measured the imputation and downstream task performance on 10% to 50% MCAR and MNAR missing values. The authors show that the proposed method outperforms the baselines, closely followed by *k*-NN and iterative *k*-NN.

We summarize the abovementioned articles and related benchmarks in Table 1. Most benchmarks use broad missingness fractions but lack realistic missingness conditions or a large number of heterogeneous datasets. Furthermore, no article systematically compares the imputation quality and impact on downstream tasks for imputation methods trained on complete and incomplete data. Studies presenting novel imputation methods based on deep learning often lack a comprehensive comparison with classical methods under realistic conditions, with few exceptions (Zhang et al., 2018). To summarize the contributions of our work, we complement existing research by providing a broad and comprehensive benchmark imputation method with respect to the following dimensions:

1) Number and heterogeneity of datasets:
We use 69 datasets with numeric and categorical columns
2) Varying downstream tasks:
We use 21 regression, 31 binary classification, and 17 multiclass classification tasks
3) Realistic missingness patterns and the amount of missing values:
We use MCAR, MAR, and MNAR missingness patterns and 1%, 10%, 30%, and 50% missing values
4) Imputation methods and optimized hyperparameters:
We use six imputation methods that range from simple baselines to modern deep generative models
5) Evaluation on imputation performance and impact on downstream task performance:
We systematically compare the imputation methods based on their imputation performance and how they impact the performance of a downstream model
6) Training on complete and incomplete data:
We simulate and compare the performance when imputation models can learn from complete and incomplete data

**TABLE 1:** An overview of related benchmarks. In contrast to our benchmark, all other studies focus on specific aspects such as downstream tasks or missingness conditions. Most importantly, no paper systematically compares imputation methods trained on complete and incomplete datasets. Abbreviations: the symbol *#* stands for the number, *B* means baselines, *Imp* means imputation quality, *Down* means impact on the downstream task, *Comp* means complete data, *Incomp* means incomplete data.

Study	# Datasets/tasks	# B	Missingness		Evaluation		Training on	
			Pattern	Fraction	Imp	Down	Comp	Incomp
Poulos and Valle (2018)	2 binary classification	6	MCAR MAR	0%, 10%, 20%, 30%, 40%	No	Yes	*Unclear*	
Jadhav et al. (2019)	5 datasets	7	*Unclear*	10%, 20%, 30%, 40%, 50%	Yes	No	*Unclear*	
Woznica and Biecek (2020)	13 binary classification	7	*Unclear* *^a^*	1%–∼33%	No	Yes	No	Yes
Zhang et al. (2018)	10 classification3 regression	11	MNAR	25%, 50%, 75%	Yes	Yes^b^	*Unclear*	
Bertsimas et al. (2017)	84 datasets (classification and regression)	5	MCAR MNAR	10%, 20%, 30%, 40%, 50%	Yes	Yes^b^	*Unclear*	
Ours	21 regression	6	MCAR MAR MNAR	1%, 10%, 30%, 50%	Yes	Yes	Yes	Yes
	31 binary classification							
	17 multiclass classification							

a Authors use incomplete datasets and, therefore, do not know the missingness pattern

b For a subset of the experiments, i. e, not systematical.

## 3 Methods

One of the main goals of this work is to provide a comprehensive evaluation of missing value imputation methods under realistic conditions. In particular, we focus on two aspects: 1) a broad suite of real-world datasets and tasks and 2) realistic missingness patterns. The following sections describe the datasets and missingness patterns we considered and the data preprocessing steps. Then follows a detailed description of the compared imputation methods, the used hyperparameter optimization strategies, and metrics for evaluation.

### 3.1 Datasets

We focus on a comprehensive evaluation with several numeric datasets and tasks (regression, binary classification, and multiclass classification). The OpenML database (Vanschoren et al., 2013) contains thousands of datasets and provides an API. The Python package scikit-learn (Pedregosa et al., 2011) can use this API to download datasets and create well-formatted DataFrames that encode the data properly.

We filter available datasets as follows. To calculate the imputation performance, we need ground truth datasets without missing values. Moreover, especially deep learning models need sufficient data to learn their task properly. However, because we plan to run many experiments, the datasets must not be too big to keep training times feasible. For this reason, we choose datasets without missing values that contain 5 to 25 features and 3.000 to 100.000 observations. We then removed duplicated, corrupted, and Sparse ARFF^2^ formatted datasets.

The resulting 69 datasets are composed of 21 regression, 31 binary classification, and 17 multiclass classification datasets. The supplementary material contains a detailed list of all datasets and further information, such as OpenML ID, name, and the number of observations and features.

### 3.2 Missingness Patterns

Most research on missing value imputation considers three different types of missingness patterns:

• Missing completely at random (MCAR, see Table 2): Values are discarded independently of any other values
• Missing at random (MAR, see Table 3): Values in column *c* are discarded depending on values in another column k≠c
• Missing not at random (MNAR, see Table 4) Values in column *c* are discarded depending on their value in *c*

**TABLE 2:** Applying the MCAR condition to column *height* discards five out of ten values independent of the height values.

Height	Height_MCAR_
179.0	?
192.0	?
189.0	189.0
156.0	156.0
175.0	?
170.0	170.0
181.0	?
197.0	?
156.0	156.0
160.0	160.0

**TABLE 3:** In the MAR condition, *height* values are discarded dependent on values in another column, here *gender*. All discarded *height* values correspond to rows in which *gender* was *male*.

Height	Gender	Height_MAR_
200.0	M	?
191.0	M	?
198.0	F	198.0
155.0	M	?
206.0	M	?
152.0	F	152.0
175.0	F	175.0
159.0	M	?
153.0	F	153.0
209.0	M	209.0

**TABLE 4:** In the MNAR condition, *height* values are discarded dependent on the actual *height* values. All discarded values correspond to small *height* values.

Height	Height_MNAR_
154.0	?
181.0	181.0
207.0	207.0
194.0	194.0
153.0	?
156.0	?
198.0	198.0
185.0	185.0
155.0	?
164.0	?

The missingness pattern most often used in the literature on missing value imputation is MCAR. Here, the missing values are chosen independently at random. Usually, the implementations of this condition draw a random number from a uniform distribution and discard a value if that random number was below the desired missingness ratio. Few studies report results on the more challenging conditions MAR and MNAR. We here aim for realistic modeling of these missingness patterns inspired by observations in large-scale real-world datasets as investigated in the work of Biessmann et al. (2018). We use an implementation proposed in the work of Schelter et al. (2020) and Schelter et al. (2021), which selects two random percentiles of the values in a column, one for the lower and the other for the upper bound of the value range considered. In the MAR condition, we discard values if values in a random other column fall in that percentile. In the MNAR condition, we discard values in a column if the values themselves fall in that random percentile range.

### 3.3 Data Preprocessing

Data preprocessing is often an essential part of ML pipelines to achieve good results (Sculley et al., 2015). In our experiments, we apply the following three preprocessing steps for all the imputation methods:

• Encode categorical columns: Categories are transformed into a numerical representation, which is defined on the training set and equally applied to the test set
• Replace missing values: To avoid the imputation model from failing
• Normalize the data: The columns are rescaled to the same range, which is defined on the training set and equally applied to the test set

However, the concrete techniques for discriminative imputation, as described in Section 3.4.1, Section 3.4.2, Section 3.4.3, and Section 3.4.4, and generative approaches, as described in Section 3.4.5, are different.

For discriminative imputation approaches, we substitute missing values with their column-wise mean/mode value, one-hot encode categorical columns, and normalize the data to zero mean and unit variance. For generative imputation approaches, we need to preserve the number of columns. For this reason, we encode the categories of categorical columns as values from 0 to n−1, where *n* is the number of categories. Then, the missing values are replaced with random uniform noise from 0 to 0.01, and finally, the data are min–max scaled ranging from 0 to 1.

### 3.4 Imputation Methods

In this section, we describe our six different imputation methods. The overall goal of an imputation method is to train a model on a dataset $X\in {\mathbb{R}}^{n\times d}=\left[x_{1},x_{2},\ldots ,x_{i-1},x_{i+1},\ldots ,x_{d}\right]$, where *d* is the number of features, *n* is the number of observations, and $x_{i}$ denotes the to-be-imputed column. To abstract crucial steps such as preprocessing the data (see Section 3.3) and cross validating the imputation method’s hyperparameters (see Section 3.5), we define a framework implemented by all of the following imputation approaches.

#### 3.4.1 Mean/Mode Imputation

As a simple imputation baseline, we use the column-wise mean for numerical or mode, i.e., the most frequent value, for categorical columns to fill missing values.

#### 3.4.2 *K*-NN Imputation

A popular ML imputation baseline is *k*-NN imputation, also known as Hot-Deck imputation (Batista and Monard, 2003). For our implementation thereof, we use scikit-learn’s KNeighborsClassifier for categorical to-be-imputed columns and KNeighborsRegressor for numerical columns, respectively.

#### 3.4.3 Random Forest Imputation

Similarl to the *k*-NN imputation approach, as described in Section 3.4.2, we implement the random forest imputation method using scikit-learn’s RandomForestClassifier and RandomForestRegressor.

#### 3.4.4 Discriminative Deep Learning Imputation

In recent years, the popularity of deep-learning–based models has increased substantially. Consequently, also the application of deep learning methods for imputation has become more popular. A query on Google Scholar for *deep learning imputation* shows that the number of publications increased from 2,110 publications in 2010 to 10,100 publications in 2020, an increase of over 470%, while the number of publications found for the term *imputation* alone actually slightly decreased from 41,700 in 2010 to 40,700 in 2020. For example, Biessmann et al. (2018) show that simple deep learning models can achieve good imputation results. To represent a range of possible DL-based imputation models, we decide to optimize the model’s architecture. For this reason, we use the AutoML^3^ library autokeras (Jin et al., 2019) to implement the discriminative deep learning imputation method. For categorical columns, we use autokeras’ StructuredDataClassifier and for numerical columns StructuredDataRegressor. Both the classes take care of properly encoding the data themselves and optimizing the model’s architecture and hyperparameters. We use max_trials=50, which means autokeras tries up to 50 different model architecture and hyperparameter combinations, and epochs=50, such that each model is trained for a maximum of 50 epochs (autokeras uses early stopping by default).

#### 3.4.5 Generative Deep Learning Imputation

All of the abovementioned approaches essentially follow the ideas known in the statistics literature as *fully conditional specification* (FCS) (van Buuren, 2018): a discriminative model is trained on all but one column as features and the remaining column as the target variable. A well-known FCS method is multiple imputation with chained equations (MICE) (Little and Rubin, 2002). FCS has the advantage to be applicable to any supervised learning method, but it has the decisive disadvantage that, for each to-be-imputed column, a new model has to be trained. Generative approaches are different in that they train just one model for an entire table. All matrix-factorization–based approaches, such as those in the work of Troyanskaya et al. (2001); Koren et al. (2009); Mazumder et al. (2010), can be thought of as examples of generative models for imputation. We do not consider those linear generative models here as they have been shown to be outperformed by the mentioned methods and focus on deep learning variants of generative models only.

Generative deep learning methods can be broadly categorized into two classes: variational autoencoders (VAEs) (Kingma and Welling, 2014)^4^ and generative adversarial networks (GANs) (Goodfellow et al., 2014). In the following, we shortly highlight some representative imputation methods based on either of these two and describe the implementation used in our experiments.

##### 3.4.5.1 Variational Autoencoder Imputation

VAEs learn to encode their input into a distribution over the latent space and decode by sampling from this distribution (Kingma and Welling, 2014). Imputation methods based on this type of generative model include those in the work of Nazábal et al. (2020); Qiu et al. (2020); and Ma et al. (2020). Rather than comparing all the existing implementations, we focus on the original VAE imputation method for the sake of comparability with other approaches. To find the best model architecture, i.e., the number of hidden layers and their sizes, we follow the approach proposed by Camino et al. (2019). We optimized using zero, one, or two hidden layer(s) for the encoder and decoder and fixed their sizes relative to the input dimension, i.e., the table’s number of columns. If existing, the encoder’s first hidden layer has 50% of the input layer’s neurons and the second layer 30%. The decoder’s sizes are vice versa for upsampling the information to the same size as the input data. The latent space is also fixed to 20% of the input dimension. For training, we use Adam optimizer with default hyperparameters, batch size of 64, and early stopping within 50 epochs.

##### 3.4.5.2 Generative Adversarial Network Imputation

GANs consist of two parts—a generator and a discriminator (Goodfellow et al., 2014). In an adversarial process, the generator learns to generate samples that are as close as possible to the data distribution, and the discriminator learns to distinguish whether an example is true or generated. Imputation approaches based on GANs include those in the work of Yoon et al. (2018); Shang et al. (2017); and Li et al. (2019). Here, we employ one of the most popular approaches of GAN-based imputation, Generative Adversarial Imputation Nets (GAIN) (Yoon et al., 2018). GAIN adapts the original GAN architecture as follows. The generator’s input is the concatenation of the input data and a binary matrix that represents the missing values. The discriminator learns to reconstruct the mask matrix. Its input is the concatenation of the generator’s output and a hint matrix, which reveals partial information about the missingness of the original data. The computation of the hint matrix incorporates the introduced hyperparameter hint_rate. A second hyperparameter *α* that GAIN introduces helps to balance the generator’s performance for observed and missing values. For training, we use Adam optimizer with default hyperparameters except for the learning rate for the generator and the discriminator, batch size of 64, and early stopping within 50 epochs.

### 3.5 Hyperparameter Optimization

Optimizing and cross validating hyperparameters are crucial to gain insights into a model’s performance, robustness, and training time. Therefore, we choose for each imputation model the, as we find, most important hyperparameters and optimize them using cross-validated grid-search. For the *k*-NN and random forest imputation methods, we use 5-fold cross validation, whereas we only 3-fold cross validate VAE and GAIN to reduce the overall training time. Table 5 gives an overview of all the imputation approaches and their hyperparameters we optimize, and the number of combinations. We do not define hyperparameter grids for mean/mode and DL imputation, as the former is parameterless and the latter is optimized by autokeras.

**TABLE 5:** An overview of all imputation methods and their hyperparameters we optimized. *Mean/mode* imputation does not have any hyperparameters, and *Discriminative DL* is optimized using autokeras, which is why we do not explicitly define a hyperparameter grid.

Imputation method	Hyperparameters		Grid size
	Name	Values	
Mean/mode	—	—	—
*k*-NN	n_neighbors	(1, 3, 5)	3
Random forest	n_estimators	(10, 50, 100)	3
Discriminative DL^a^	—	—	—
VAE	n_hidden_layers	(0, 1, 2)	3
GAIN	alpha	(1, 10)	16
	hint_rate	(0.7, 0.9)	—
	generator_learning_rate	(0.0001, 0.0005)	—
	discriminator_learning_rate	(0.00001, 0.00005)	—

a Optimized using autokeras, see Section 3.4.4.

### 3.6 Evaluation Metrics

To evaluate our experiments, we use two metrics: root mean square error (RMSE) and macro F1-score. The RMSE is defined as

$$RMSE=\sqrt{\frac{1}{N}\overset{N}{\underset{i=0}{\sum }}{\left(y_{i}-\hat{y_{i}}\right)}^{2}},$$ (1)

where *N* is the number of observations, $y_{i}$ is the observed values, and ${\hat{y}}_{i}$ is the predicted values. The macro F1-score is defined as the mean of class-wise F1-scores:

$$macro\;F1=\frac{1}{C}\overset{C}{\underset{i=0}{\sum }}F1_{i},$$ (2)

where *i* is the class index, *C* is the number of classes, and the definition of F1 is

$$F1=\frac{TP}{TP+\frac{1}{2}\left(FP+FN\right)},$$ (3)

where TP is the number of true positives, FP is the number of false positives, and FN is the number of false negatives.

Imputing categorical columns can be seen as a classification task. Accordingly, we measure performance in this case and for downstream classification tasks by the macro F1-score. In the following, we use the terms F1-score and F1 synonymously for macro F1-score. For regression tasks and imputing numerical columns, we use the RMSE. Since F1 is a score measure, larger values imply better performance. On the other hand, RMSE is an error measure: a smaller value indicates better performance.

## 4 Implementation and Experiments

In this section, we describe our benchmark suite in detail and its implementation.

As described in Section 3.4, we define a framework that provides for each of the six implemented imputation approaches a common API with the methods fit and transform. Fit trains the imputation model on given data while cross-validating a set of hyperparameters, and transform allows imputing missing values of the to-be-imputed column the imputation model is trained on. For our implementation, we use tensorflow version 2.4.1, scikit-learn version 0.24.1, and autokeras version 1.0.12.

The Python package jenga^5^ (Schelter et al., 2021) provides two features we use to implement our experiments. First, it implements the mechanisms to discard values for the missingness patterns MCAR, MAR, and MNAR, as described in Section 3.2. Second, it provides a wrapper for OpenML datasets, creates an 80/20 training-test split, and can automatically train a *baseline model* for the downstream task defined by the dataset. We use the default task settings of jenga in which scikit-learn’s SGDClassifier is used for classification and SGDRegressor for regression tasks. As preprocessing steps, it first replaces missing values with a constant, and second, one-hot encodes categorical columns and normalizes numerical columns to zero mean and unit variance. Finally, to train a robust model, it 5-fold cross validates the hyperparameters *loss*, *penalty*, and *alpha* using grid search. Jenga reports the baseline model’s performance (F1 for classification, RMSE for regression) on the test set.

### 4.1 Experimental Settings

Our experimental settings are listed in Table 6. Each experiment is executed three times, and the average performance metrics are reported.

**TABLE 6:** Overview of our experimental settings. We focus on covering an extensive range of the dimensions described in Section 2. In total, there are 4,968 experiments, which we repeat three times to report the mean imputation/downstream score.

Parameter	Values
Datasets	69 (see Supplementary Material)
Imputation methods	Mean/mode, *k*-NN, random forest, DL, GAIN, VAE
Missingness patterns	MCAR, MAR, MNAR
Missingness fractions	1%,10%,30%,50%

For each of the datasets, we sample one to-be-imputed column upfront, which remains static throughout our experiments.

We split the experiments into four parts. In *Experiment 1*, we compare imputation approaches with respect to their imputation quality (Section 4.1.1), and in *Experiment 2*, we compare imputation methods with respect to the impact on downstream tasks (Section 4.1.2). Both experiments are repeated in two application scenarios: *Scenario 1* (with complete training data, see Section 4.1.3) and *Scenario 2* (with incomplete training data, see Section 4.1.4).

#### 4.1.1 Experiment 1: Imputation Quality

With this experiment, we aim to reveal how accurately the imputation methods can impute the original values. With the help of jenga, we spread the desired number of missing values across all the columns of the test set. For a certain missingness pattern and fraction, e.g., 30% MAR, we introduce $\frac{30\text{\%}}{N}$ missing values of this pattern to each of the *N* columns. The evaluation of the imputation quality is then performed using the to-be-imputed column’s discarded values as ground truth and the imputation model’s predictions. If the to-be-imputed column is categorical, we report the F1-score, and for numerical columns, the RMSE.

Since this work focuses on point estimates of imputed values, the assessment of the inherent uncertainty of imputed values is beyond the scope of this evaluation. We are aware of this limitation and use a second experiment to avoid relying on these single-value summaries. Explanations and other directions to overcome those limitations are, e.g., provided by Wang et al. (2021).

#### 4.1.2 Experiment 2: Impact on the Downstream Task

In *Experiment 2*, we evaluate the impact of the different imputation approaches on numerous downstream ML tasks. For discriminative models, it is necessary to train one imputation model for each column with missing values. This fact, combined with our large number of experimental conditions (see Table 6), results in vast computational costs. To reduce those, while covering all relevant experimental conditions, we decided to discard values only in the test sets’ to-be-imputed column.

To summarize, the entire experimental procedure is as follows:

1) We train the baseline model of the downstream ML task on the training set and report its baseline score (F1 for classification and RMSE for regression tasks) on the test set
2) After discarding values in the to-be-imputed column, we again use the trained baseline model and calculate its score on the incomplete test set, hence the name incomplete
3) We then impute the missing values of the test set and, once more, using the trained baseline model, calculate the imputed score
4) Finally, we report the impact on the downstream task’s performance as the percent change of the imputation over the incomplete data relative to the baseline performance on fully observed test data:

$$impact\;on\;downstream\;task=\frac{imputed-incomplete}{baseline}$$ (4)

#### 4.1.3 Scenario 1: Training on Complete Data

ML researchers commonly use complete (or fully observed) data to train, tune, and validate their ML applications. This is a reasonable assumption as the quality of the training data can be controlled better than that of the test data when the model is deployed in production. For instance, one can use crowdsourced tasks to collect all necessary features in the training data or use sampling schemes that ensure complete and representative training data. In this scenario, one can easily train an imputation model on complete data and use it to impute missing values in the test data before it is fed into the downstream ML model. We use *Scenario 1* to simulate such situations and run both experiments, as described in Section 4.1.1 and Section 4.1.2.

#### 4.1.4 Scenario 2: Training on Incomplete

Another common scenario is that not only the test data but also the training data have missing values. Thus, the imputation and downstream ML model has to be trained on incomplete training data. Also, in this scenario, we should expect missing values in the test data, which have to be imputed before applying the downstream ML model. To evaluate this application scenario, we adapt *Experiment 1* and *Experiment 2* slightly.

We first introduce missing values in the training and test set and then train the baseline and imputation models based on these incomplete data. The calculation of the imputation quality (*Experiment 1*, Section 4.1.1) remains the same. However, to calculate the impact on the downstream task, we lack the availability of the baseline score on complete data. Therefore, we adapt Eq. 4 by replacing the baseline denominator with incomplete. That means, in this scenario, we report the percent change of the imputation over the incomplete data relative to the downstream task performance on incomplete data:

$$impact\;on\;downstream\;task=\frac{imputed-incomplete}{incomplete}.$$ (5)

## 5 Results

In this section, we describe and visualize the results of our experiments. For the visualization, we choose to use box plots for all four experiments/scenarios. These allow us to get a decent impression of the distribution of the results based on quantiles. In contrast, the confidence bands of line charts would overlap too much to derive meaningful interpretations. The vertical split represents the increasing difficulty for the missingness patterns: MCAR, MAR, and MNAR. To show different effects of imputing categorical or numerical columns, we further split the plots horizontally. Because we randomly sample on target column for each dataset, there are about 13% categorical (9) and 87% numerical (60) columns. Respectively, for the second experiment, the horizontal split presents classification and regression downstream tasks, which are also imbalanced: 48 classification (∼70%) and 21 regression tasks (∼30%).

### 5.1 Experiment 1: Imputation Quality

In this experiment, we evaluate the imputation performance of each method when training on complete data. As described above, our goal was to provide a broad overview of the imputation methods’ performance on various datasets. Using randomly sampled to-be-imputed columns on heterogeneous data leads to a wide range of values for their evaluation metric (F1/RMSE), making it difficult to compare. To solve this problem, we split the results into categorical and numerical imputations and compute the rank for each imputation method, missingness pattern, and fraction combination separately. Since we use six imputation methods, there are six ranks, where rank 1 is the best and rank 6 the worst. If two or more methods perform equally, we assign the same rank. Imputation methods that failed to train the model get rank 6. For each experimental setting and every dataset, we have ordered ranks for the imputation methods. This allows us to interpret the results relative to each other. For example, if one imputation method ranks best, i.e., rank 1, for all datasets, we know that all other imputation methods have at least rank 2.

#### 5.1.1 Scenario 1: Training on Complete Data

**FIGURE 1:**
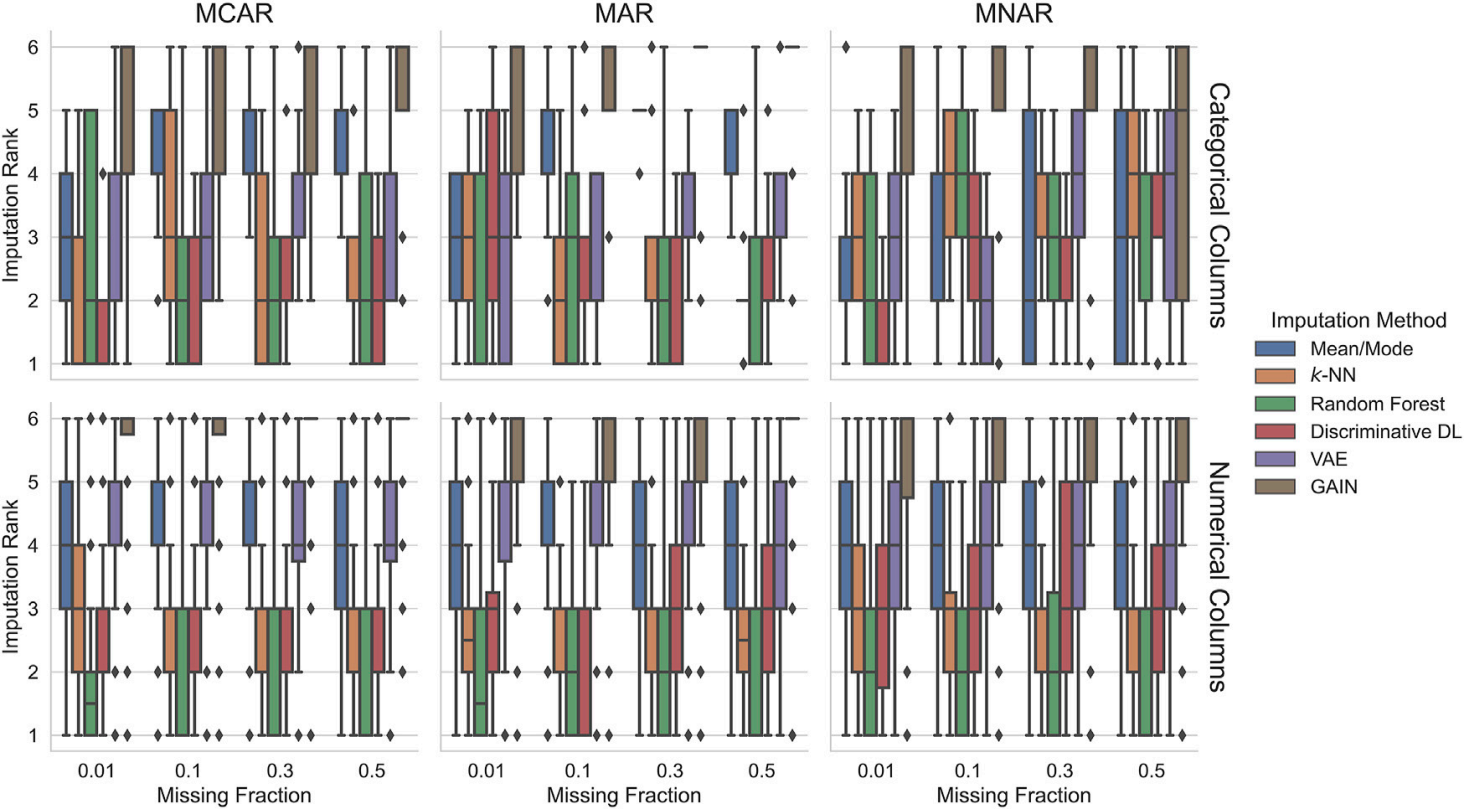
Imputation ranks of the imputation methods trained on complete data. Ranks are computed for each experimental condition characterized by the dataset, missingness pattern, and missingness ratio. Since we compare six imputation methods, the possible imputation ranks range between 1 and 6. In most conditions, random forest, *k*-NN, and discriminative DL perform best. Generative deep learning methods tend to perform worst. In the most challenging MNAR condition, mean/mode imputation achieves competitive results.

Figure 1 presents the imputation results when training on complete data. In about 33% of these results, GAIN failed during training and got assigned the worst rank six. Investigating the errors reveals that GAIN’s discriminator loss gets NaN at some point, leading to failures on further calculations and a failing training process. This depends heavily on the discriminator’s learning rate and the dataset. GAN-based models are generally known as hard to train, which is why improvements for training GANs are introduced to make their training process more robust, e.g., the work of Salimans et al. (2016); Heusel et al. (2017); and Miyato et al. (2018). However, optimizing the hyperparameters for all datasets is out of the scope of this article. Therefore, we decide to define the hyperparameter grids once and incorporate the imputation methods’ robustness regarding their hyperparameters into our evaluation.

When imputing categorical columns, there is no clear best method. However, in many settings, the discriminative DL approach achieves in 75% of the cases at least rank three or better. Very similar but slightly worse results are shown by the random forest imputation method. For MCAR with 50% missing values and MAR with 10% to 50% missingness, the *k*-NN imputation approach performs well and gets for 75% of the cases at least rank three or better. VAE achieves in 50% of the cases a rank between two and four. GAIN shows in most settings consistently the worst performance: rank four or worse in 75% of the cases. Interestingly, mean/mode imputation scores better rank for the more complex settings with MNAR missingness pattern.

When imputing numerical columns, the differences are more pronounced. Random forest is the only method that achieves one of the first three ranks in 75% of the cases throughout all the experimental conditions. Also, *k*-NN shows good results, ranking second or third in most settings in 50% of the cases. Very similar results are achieved by the discriminative DL method that tends to lose performance from MAR with 30% missingness to MNAR with 50% missing values. Again VAE ranges most of the time between ranks three and five, similar to mean/mode imputation, and GAIN gets the worst ranks five and six.

To summarize, simple imputation methods, such as *k*-NN and random forest, often perform best, closely followed by the discriminative DL approach. However, for imputing categorical columns with MNAR missing values, mean/mode imputation often performs well, especially for high fractions of missing values. The generative approaches get middle ranks (VAE) or range on the worst ranks (GAIN).

#### 5.1.2 Scenario 2: Training on Incomplete Data

**FIGURE 2:**
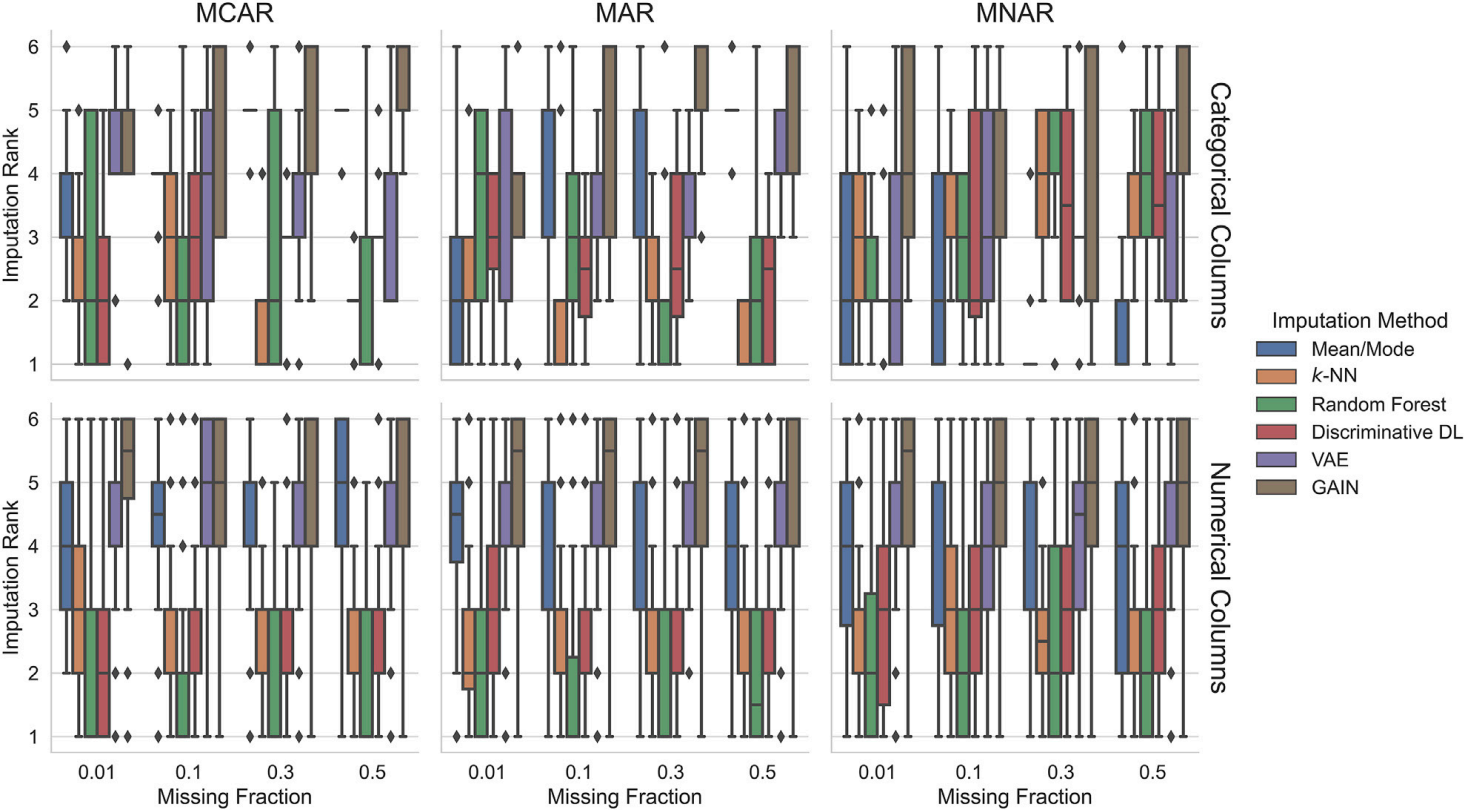
Imputation ranks of the imputation methods trained on incomplete data. Ranks are computed for each experimental condition characterized by the dataset, missingness pattern, and missingness ratio. Since we compare six imputation methods, the possible imputation ranks range between 1 and 6. Similar to the training on fully observed data random forest, *k*-NN and discriminative DL perform better than generative deep learning methods in most settings. In the MNAR conditions, the imputation quality of all the imputation approaches degrades in favor of mean/mode that outperforms the other for 30% and 50% missingness.

Figure 2 shows the imputation performance in *Scenario 2*, i.e., when training on incomplete data. Imputing categorical columns with increasing difficulty, the ranks of mean/mode imputation improve. From MCAR 30% to MNAR 50%, *k*-NN is in 75% of the cases on at least the third rank or better, and often, it ranges on the first and second rank. For MNAR, its performance degrades gradually in favor of mean/mode that shows surprisingly good results, especially for the most challenging settings (MNAR with 30% and 50% missing values) where it outperforms others in at least 75% of the cases. Random forest has very high variance, but on most missingness fractions with MCAR pattern, it ranks in 50% of the cases on rank two or better. For MNAR, its rank improves with higher missingness fractions, whereas this trend reverses for MAR. In most cases, the generative methods rank worst (GAIN) and on the middle ranks (VAE). However, with high missingness and when missing values are MNAR, they can perform better.

Similar to the fully observed training case (Section 5.1.1), imputation on numerical columns yields a clearer ranking than for categorical missing values. The imputation methods *k*-NN and random forest rank best with a tendency of random forest to outperform *k*-NN, where random forest’s variance is higher. The discriminative DL approach yields a very similar performance to the *k*-NN for the MCAR and MAR settings. In the more challenging MNAR setting, it ranks slightly worse. For MCAR, mean/mode imputation ranks in almost all settings in 50% of the cases between ranks four and five and for MAR and MNAR, between ranks three and five. Again the generative methods rank in almost all settings in 75% of the cases worse than rank four, where VAE seldom ranks worst.

Overall, *Scenario 1* (Figure 1) and *Scenario 2* (Figure 2) results for numerical columns are very similar. GAIN has become better in *Scenario 2*, although it still ranks worst. For categorical columns, generally, the ranks show higher variance. Most imputation methods worsen when the experimental settings’ difficulty is higher, especially for MNAR, except for mean/mode, which ranks better for MNAR. This effect is even explicit when training on incomplete data. Generally, using methods such as *k*-NN or random forest achieves best results in most settings and cases.

### 5.2 Experiment 2: Impact on the Downstream Task

In this experiment, we evaluate the imputation method’s impact on the downstream performance in two scenarios: the imputation model was trained on complete and incomplete data. As described in Section 4.1.2, this time, we discard only values in the dataset’s randomly sampled target column.

#### 5.2.1 Scenario 1: Training on Complete Data

**FIGURE 3:**
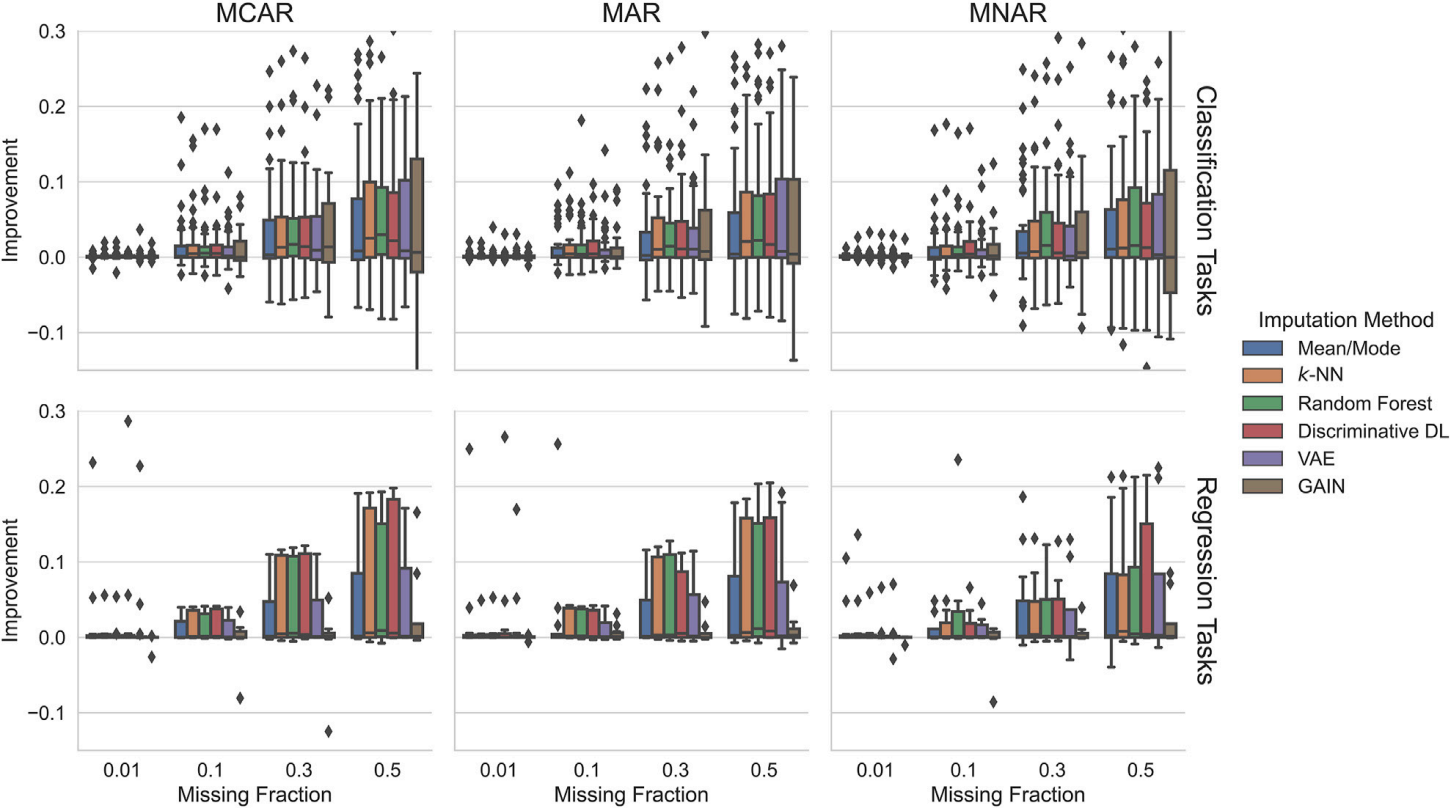
Does imputation on incomplete test data improve predictive performance of a downstream ML model? We plot the improvement of the downstream ML model after imputation with imputation models trained on fully observed data. The downstream performance is compared to the performance obtained on incomplete test data, normalized by the ML model performance on fully observed test data. Overall, the classical ML methods and discriminative DL perform best achieving relative improvements of up to 10% and more relative to fully observed test data.

Since training GAIN failed in about 33% of the experiments (see Section 5.1.1), we exclude those from this evaluation. Figure 3 visualizes how much the predictive performance of a downstream ML model improves compared to incomplete test data and normalized by the downstream performance obtained on fully observed test data (Eq. 4). This metric is labeled *Improvement* and represented on the plots’ y-axis.

In all cases, using imputation approaches increases the downstream performance in 75% of the cases. Not surprisingly, independent of the downstream task and the missingness pattern, the more the missing values exist, the better the potential improvement, shown by the method’s increasing median and 75% quantile.

For regression tasks, all imputation methods on all settings degrade the performance in less than 25% of the cases. Furthermore, they hold great potential for improving the performance in the range of ∼10% and ∼15% for 30% and 50% MCAR or MAR missing values. However, there is a tendency from MCAR to MNAR that the potential performance degrades. In most settings, random forest’s median improvement is the best, followed by *k*-NN and discriminative DL. This effect also holds for their potential improvement (75% quantile), except for 50% MNAR, where it is about five percentage points higher than the others. In most settings, VAE and mean/mode increase the downstream performance very similar but worse than the other three, and GAIN is always the worst.

For classification tasks, few imputation methods in some settings show degrading performance in slightly more than 25% of the cases. However, their median imputation performance is always positive and generally higher than for regression tasks. In general, the potential improvements of the methods are in all settings roughly the same. As for regression tasks, random forest, followed by *k*-NN and discriminate DL, hold in 50% of the cases the best performance. Unfortunately, this degrades from MCAR to MNAR. Surprisingly, this time, GAIN holds much more potential improvement and performs in many settings better than VAE, especially when the missingness fraction is high.

All in all, independent of the experimental settings, random forest performs in 50% of the cases best, closely followed by *k*-NN and discriminative DL. In general, when using imputation, the expected improvement is for classification higher than for regression tasks. This effect also holds for the missingness fractions: the higher the missingness fraction, the higher the potential improvements. Only in less than 25% of all cases, we found degraded downstream performance.

#### 5.2.2 Scenario 2: Training on Incomplete Data

**FIGURE 4:**
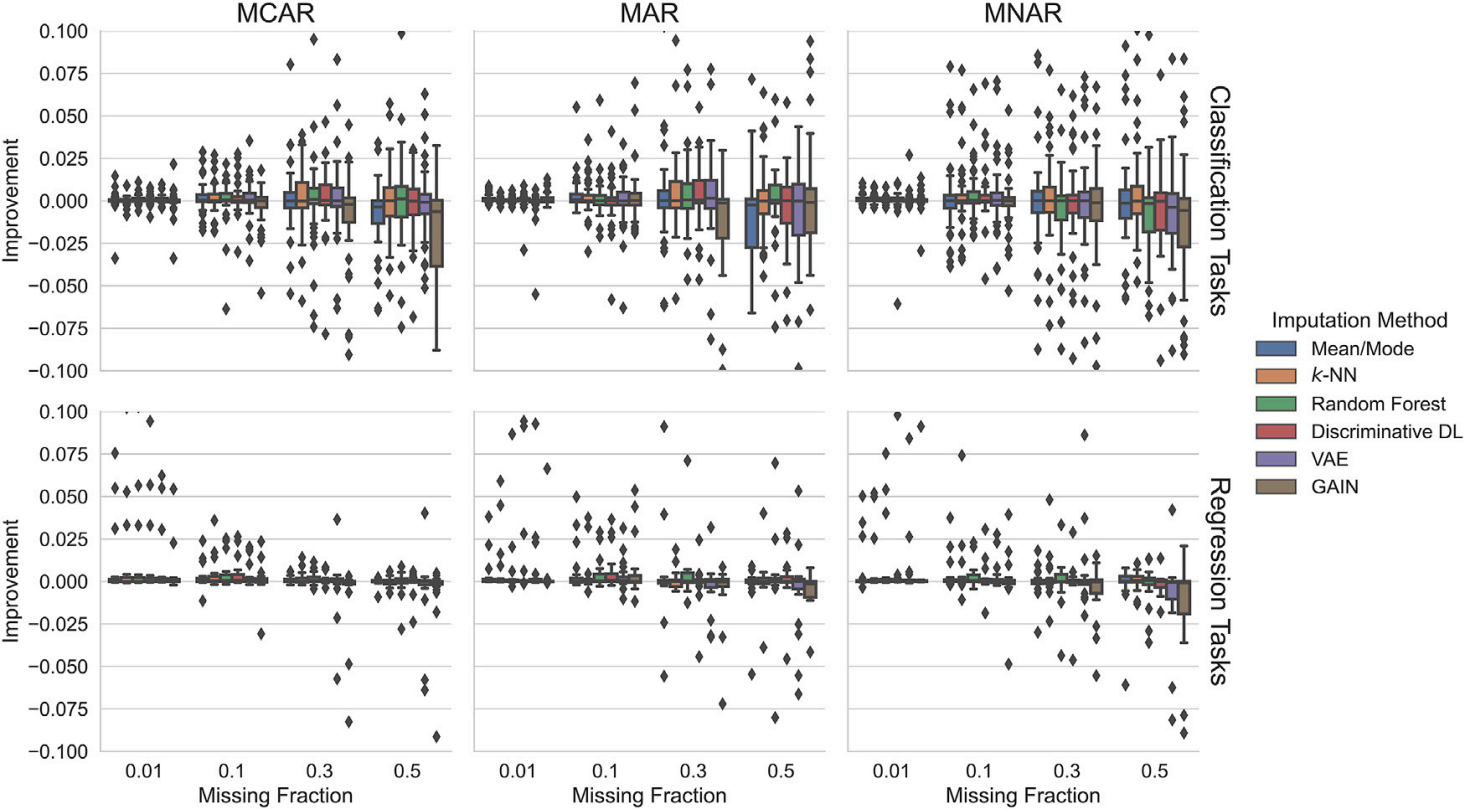
Impact on the downstream task of the six imputation methods trained on incomplete data. In regression tasks, no considerable improvements are achieved. In some cases, imputation worsened the downstream ML model. In classification tasks, in contrast, we observe slightly positive effects in some settings, but negative effects predominate in the harder settings.

Figure 4 illustrates the impact imputation has on the downstream task. We show how many percent the predictive performance of a downstream ML model improves compared to incomplete test data. This metric is labeled *Improvement* and represented on the plots’ y-axis. Here, the different scaling must be taken into account, i.e., the relative improvements are considerably smaller compared to the first scenario. One reason for this is the different basis for calculating the relative values (see Sections 4.1.2 and Sections 4.1.4).

The potential improvements when the imputation methods are trained on incomplete data are marginal. In all settings, there are hardly any improvements greater than 1%. However, with 30% missing values or fewer, most cases have a positive impact.

For classification tasks with up to 30% MCAR or MAR missingness, there, for all imputation methods, but GAIN, are mostly very small but positive improvements, where higher missing fractions yield potentially higher improvements (75% quantile). However, high missingness fractions shift the improvements into the negative range, i.e., degrade the performance. For MNAR only for 1% and 10% missing values, we see mostly improvements, and for 30% or 50% missingness, the downstream performance degrades in most cases.

For regression tasks, there are hardly any potential improvements over 0.5%. On the other hand, there are also much fewer cases where imputation potentially degrades the performance. Outstanding is random forest, which yields in most settings the highest performance and the generative approaches that harm the performance when missingness is 30% or higher.

To summarize, for up to 30% missing values independent of the missingness pattern or downstream tasks, imputation increases the performance in most cases. Using random forest holds the best chance in almost all settings to improve the downstream performance.

### 5.3 Computational Complexity

Our results demonstrate that simple ML methods are often on par with modern deep learning methods. An important question in this context is how the various methods compare in terms of their computational complexity: if methods yield similar predictive performance, it is preferable to use those alternatives with the least computational effort. To measure the training and inference time, we use a subset of our experiments: all datasets, missingness fractions, and imputation methods (shown in Table 6) with MCAR pattern. We first train the imputation method on complete data, then discard the values of the given missingness fraction in the training set, and impute those missing values. The wall-clock run time is measured in seconds when calling our framework’s fit and transform methods (see Section 4 for details), which means that the training duration incorporates hyperparameter optimization (see Section 3.5 for details).

Because training and inference time depends heavily on the dataset’s size, directly averaging all experiments for the imputation methods leads to very similar mean but extremely high standard deviation values. For this reason, we first compute the mean duration and the standard deviation relative to its mean separately for training and inference for the imputation methods on each dataset. Second, we average those values for each imputation method and present them in Table 7. Using this approach helps to average overall experiments and, at the same time, gives indicators for the training and inference durations, as well as their variance.

**TABLE 7:** Training and inference duration for each imputation method in seconds. We use the wall-clock run time to measure the durations for training, including hyperparameter optimization and inference for all datasets with MCAR missingness pattern and all fractions shown in Table 6. Because training and inference durations depend heavily on the dataset size, we first calculate the durations’ mean and relative standard deviation for each imputation method on every dataset. Second, we average those mean durations and relative standard deviations for the imputation methods and present them as *Mean duration* and *Rel. SD* separately for *Training* and *Inference*.

Imputation method	Training		Inference	
	Mean duration	Relative standard deviation	Mean duration	Relative standard deviation
Mean/mode	0.005	0.550	0.029	0.171
*k*-NN	41.204	0.254	7.018	0.602
Random forest	226.077	0.119	24.048	0.236
Discriminative DL	6,275.019	0.405	440.389	0.211
VAE	71.095	0.099	11.215	0.085
GAIN	878.058	0.312	137.966	0.083

As expected, if the imputation model’s complexity increases, their training duration increases too, most of the time by multiple factors. There are two exceptions: discriminative DL and VAE, and an explanation for this could be their number of hyperparameter combinations optimized during training. VAE optimizes only three, GAIN 16 and discriminative DL 50 combinations, representing their training durations order.

Similarly, the inference time increases with the model’s complexity. The differences are clear but not as high as for the training durations. Higher inference standard deviations, e.g., for *k*-NN and random forest (and discriminative DL), indicate that the best hyperparameters found strongly vary with the experimental settings and influence the model’s computational complexity for inference. One reason for the discriminative DL’s and GAIN’s high training standard deviations could be the usage of early stopping and, at the same time, indicate that it is important to try a huge number of hyperparameters to achieve good results. For mean/mode, the high standard deviation is likely an artifact of the very small training duration. Changes in milliseconds for computations are common and represent a large change relative to the mean/mode imputation’s mean duration.

To summarize, the increasing complexity of the imputation methods is represented in their training and inference duration. For training more complex models, this is supported by a higher variance of training time, indicating the necessity to try a wide range of hyperparameters. On the other hand, once found, the hyperparameters for generative models influence the inference time less than for *k*-NN or random forest, whose prediction times depend heavily on the hyperparameters.

## 6 Discussion

We investigated the performance of classical and modern imputation approaches on a large number of heterogeneous datasets under realistic conditions. In the following, we highlight some of the key findings.

### 6.1 Simpler Imputation Methods Yield Competitive Results

When evaluating imputation quality, our results demonstrate that simple supervised learning methods achieve competitive results and, in many cases, outperform modern generative deep-learning–based approaches. In particular, in the MCAR and MAR settings, we see in Figures 1, 2 that *k*-NN, random forest, and the discriminative DL approach are, for at least 50% of the cases, among the better ranks one, two, or three. Random forest tends to achieve the best rank more often. This effect is largely independent of whether the imputation methods are trained on complete or incomplete data.

This finding is in line with the work of Poulos and Valle (2018); Jadhav et al. (2019); and Bertsimas et al. (2017). In these previous studies, the authors report that *k*-NN imputation is the best choice in most situations. However, Jadhav et al. (2019) and Bertsimas et al. (2017) did not incorporate a random forest imputation method. Other comparisons show a slight advantage of discriminative deep learning methods over random forests (Biessmann et al., 2019), but these experiments were conducted on a much smaller selection of datasets.

For categorical columns (see Figures 1, 2, upper row) in the more challenging imputation settings MAR or MNAR with large missingness fractions, the mean/mode imputation tends to achieve better ranks. This effect can be attributed to the fact that the sets of observed categorical values often have small cardinality. Especially for skewed distributions, using the most frequent value to substitute missing values is a good approximation of the ground truth. If the training data contains a large fraction of missing values, the underlying dependencies exploited by learning algorithms are difficult to capture. For this reason, mean/mode scores for higher MNAR missing values in 75% of the cases are on rank two or better (visualized in Figure 2). Poulos and Valle (2018) did not explicitly calculate the ranks, but their plots show the same tendency.

Since GAIN failed in about 33% of settings when training data were complete, this could be a reason why, in most cases, GAIN achieves the worst ranks (see Figure 1). This is supported by the fact that GAIN does not fail for settings with incomplete training data and often shows better ranks (see Figure 2).

All in all, using random forest, discriminate DL, or *k*-NN is a good choice in most experimental settings and promises the best imputation quality. However, incorporating the model’s training and inference time, presented in Table 7, shows that the discriminative DL approach is substantially slower for training and inference than the other two methods. This is because we used the expensive default model optimization of AutoKeras. Exploring fewer hyperparameters could decrease its imputation performance drastically. The training duration’s high variance indicates that trying a large number of hyperparameters is necessary for good performance because early stopping would finish the training if the model converges. *k*-NN’s standard deviation for inference is in contrast to random forest’s very high standard deviation. This is expected as the inference time grows exponentially with the number of training data points. We conclude that given the similar performance of *k*-NN and random forests when the training dataset is large, random forests (or similar methods) should be preferred over naive *k*-NN implementations. Alternatively, one might use appropriate speedups for the nearest-neighbor search, such as kd-trees or approximate nearest-neighbor search.

To summarize, the best performing imputation approach is random forest. It not only ranks best in most experimental settings but also shows a good balance of training, including optimizing hyperparameters and inference time that is not influenced by the training set size. However, when coping with datasets that miss 30% or more values of the pattern MNAR, imputing categorical columns with their mode compares favorably with more sophisticated imputation approaches. Our results demonstrate that, especially in the challenging scenarios where a large fraction of values is missing, there is a high variance in the imputation performance metrics. This shows that, in these experimental settings, we cannot conclude that one method is consistently worse than others. But all in all, our results suggest that high-capacity deep learning models are not better than conventional methods like random forests and generative models are not consistently better than discriminative models.

### 6.2 Substantial Downstream Improvements When the Imputation Method Was Trained on Complete Data

Our results show that imputation can have a substantial positive impact on predictive performance in downstream ML tasks. We observe improvements in the downstream task of 10–20% in more than 75% of our experiments. This holds for most imputation methods; we did not observe a clear advantage for an imputation method overall. Taking into account the considerable differences in wall-clock run time, our results indicate that also when choosing an imputation method that is both fast and improves downstream predictive performance random forests would be the preferred imputation method.

The positive impact of imputation on downstream performance is most pronounced when the imputation methods were trained on fully observed data. When imputation methods were trained on incomplete data, the positive impact of imputing missing values in the test data was substantially lower, sometimes even negative. While this might seem a disadvantage, we emphasize that, in many application use cases, we can ensure that the training data be fully observed, for instance, by acquiring more data before training the imputation and the downstream ML model.

### 6.3 Limitations

Because one of the main goals of this study is a comprehensive comparison of imputation methods on a large number of datasets and missingness conditions, we made some decisions that limit our results.

First, we focus on point estimates of imputed values rather than multiple imputations because it is 1) easier to handle in automated pipelines and 2) can be considered a more relevant scenario in real-world applications of imputation methods. Thus, we do not consider the inherent uncertainty of the imputation process. We decided to measure and compare the impact imputation methods have on the downstream performance instead of using an evaluation framework that explicitly evaluates the uncertainties, e.g., proposed by Wang et al. (2021). However, comparing imputation methods with respect to the calibration of their uncertainty estimates is an important topic for future research and could be conducted with the same experimental protocol that we developed for our point estimate comparisons.

Second, the used datasets consist of a maximum of 25 features and 100k observations. For this reason, we cannot conclude from our experiments how the imputation methods perform on large-scale datasets. Furthermore, our datasets only contain numerical or categorical columns and no image- or text-based data, e.g., used in other deep-learning–based imputation approaches (Biessmann et al., 2018). However, in that work, the authors only considered text data as an input field to an imputation method, not as a column that could be imputed. Generally, most modern ML applications that involve text data are based on rather sophisticated natural language models. Combinations of such models with tabular data are an important field of research (Yin et al., 2020) but beyond the scope of most imputation research so far.

Third, to measure the imputation impact on the downstream performance, we discarded and imputed values in only a single column. Therefore, the impact depends heavily on the chosen column’s importance (e.g., see the work of Schelter et al. (2021)). Generally, the impact when using an imputation model could vary when multiple columns are affected by missing values.

## 7 Conclusion

In this study, we developed an experimental protocol and conducted a comprehensive benchmark for imputation methods comparing classical and modern approaches on a large number of datasets under realistic missingness conditions with respect to the imputation quality and the impact on the predictive performance of a downstream ML model. We also evaluated how the results changed when the imputation and downstream model were trained on incomplete data.

Our results can be summarized in two main findings. First, we demonstrate that imputation helps to increase the downstream predictive performance substantially regardless of the missingness conditions. When training data are fully observed, our results demonstrate that, in more than 75% of our experiments, imputation leads to improvements in downstream ML model predictive performance between 10% and 20% for classification tasks and around 15% for regression tasks. We conclude that when training data are fully observed, an imputation model should be trained along with the downstream ML model to improve data quality problems in the data ingested at inference time by a downstream ML component.

Second, we find that, in almost all experiments, random-forest–based imputation achieves the best imputation quality and the best improvements on the downstream predictive performance. This finding is in line with previous imputation benchmark research in more constrained experimental conditions (see also Section 2). Yet, some aspects of these results appear at odds with some recent work on deep learning methods. While we are aware of the limitations of our experiments (see also Section 6.3), we are convinced that the experimental protocols developed in this study can help to test imputation methods better and ultimately help to stress test these methods under realistic conditions in large unified benchmarks with heterogeneous datasets (Sculley et al., 2018; Bender et al., 2021).

## Data Availability

Publicly available datasets were analyzed in this study. These data can be found here: https://www.openml.org.

## Footnotes

1 A great example from life sciences is given in the work of Ziemann et al. (2016).

2 Attribute-relation file format.

3 “Automated machine learning (AutoML) (…) automatically set [the model’s] hyperparameters to optimize performance,” Hutter and Frank (2019).

4 We focus on probabilistic autoencoders here as there are more imputation methods available for VAEs.

5 Software package “to study the effects of common data corruptions (e.g., missing values and broken character encodings) on the prediction quality of ML models.” Source: https://github.com/schelterlabs/jenga

## References

[B1] Abedjan Z. Chu X. Deng D. Fernandez R. C. Ilyas I. F. Ouzzani M. (2016). Detecting Data Errors. Proc. VLDB Endow. 9, 993–1004. 10.14778/2994509.2994518

[B2] Abedjan Z. Golab L. Naumann F. Papenbrock T. (2018). Data Profiling. Synth. Lectures Data Manag. 10, 1–154. 10.2200/s00878ed1v01y201810dtm052

[B3] Batista G. E. A. P. A. Monard M. C. (2003). An Analysis of Four Missing Data Treatment Methods for Supervised Learning. Appl. Artif. Intelligence 17, 519–533. 10.1080/713827181

[B4] Baylor D. Breck E. Cheng H.-T. Fiedel N. Foo C. Y. Haque Z. Haykal S. Ispir M. Jain V. Koc L. Koo C. Y. Lew L. Mewald C. Modi A. N. Polyzotis N. Ramesh S. Roy S. Whang S. E. Wicke M. Wilkiewicz J. Zhang X. Zinkevich M. (2017). “Tfx,” in Proc. ACM SIGKDD Int. Conf. Knowl. Discov. Data Min., New York, NY, USA (Association for Computing Machinery), 1387–1395. Part F1296. 10.1145/3097983.3098021

[B5] Bender E. M. Gebru T. Mcmillan-Major A. Shmitchell S. Shmitchell S.-G. (2021). “On the Dangers of Stochastic Parrots,” in FAccT '21: 2021 ACM Conference on Fairness, Accountability, and Transparency, Canada (Association for Computing Machinery), 610–623. 10.1145/3442188.3445922

[B6] Bertsimas D. Pawlowski C. Zhuo Y. D. (2017). From Predictive Methods to Missing Data Imputation: An Optimization Approach. J. Mach. Learn. Res. 18 (196), 1–39.

[B7] Biessmann F. Golebiowski J. Rukat T. Lange D. Schmidt P. (2021). “Automated Data Validation in Machine Learning Systems,” in Bulletin of the IEEE Computer Society Technical Committee on Data Engineering.

[B8] Biessmann F. Rukat T. Schmidt P. Naidu P. Schelter S. Taptunov A. (2019). Datawig: Missing Value Imputation for Tables. J. Machine Learn. Res. 20, 1–6.

[B9] Biessmann F. Salinas D. Schelter S. Schmidt P. Lange D. (2018). “"Deep" Learning for Missing Value Imputationin Tables with Non-numerical Data,” . in Int. Conf. Inf. Knowl. Manag. Proc, Torino Italy (ACM Press), 2017–2026. 10.1145/3269206.3272005

[B10] Böse J.-H. Flunkert V. Gasthaus J. Januschowski T. Lange D. Salinas D. (2017). Probabilistic Demand Forecasting at Scale. Proc. VLDB Endow. 10, 1694–1705. 10.14778/3137765.3137775

[B11] Camino R. Hammerschmidt C. A. State R. (2019). Improving Missing Data Imputation with Deep Generative Models. ArXiv abs/1902, 10666.

[B12] Hutter F. Frank (2019). Automated Machine Learning - Methods, Systems, Challenges (Cham, Switzerland: The Springer Series on Challenges in Machine Learning (Springer). 10.1007/978-3-030-05318-5

[B13] Goodfellow I. Pouget-Abadie J. Mirza M. Xu B. Warde-Farley D. Ozair S. (2014). “Generative Adversarial Nets,”. Advances in Neural Information Processing Systems. Editors Ghahramani Z. Welling M. Cortes C. Lawrence N. Weinberger K. Q. (Montréal, Canada: Curran Associates, Inc.), 27, 2672–2680.

[B14] Heusel M. Ramsauer H. Unterthiner T. Nessler B. Hochreiter S. (2017). “Gans Trained by a Two Time-Scale Update Rule Converge to a Local Nash Equilibrium,” in Advances in Neural Information Processing Systems 30: Annual Conference on Neural Information Processing Systems, Long Beach, CA, USA, 2017. Editors Guyon I. von Luxburg U. Bengio S. Wallach H. M. Fergus R. Vishwanathan S. V. N. Garnett R., 6626–6637.

[B15] Jadhav A. Pramod D. Ramanathan K. (2019). Comparison of Performance of Data Imputation Methods for Numeric Dataset. Appl. Artif. Intelligence 33, 913–933. 10.1080/08839514.2019.1637138

[B16] Jin H. Song Q. Hu X. (2019). “Auto-keras: An Efficient Neural Architecture Search System,” in Proceedings of the 25th ACM SIGKDD International Conference on Knowledge Discovery & Data Mining, Anchorage AK USA (ACM), 1946–1956.

[B17] Kingma D. P. Welling M. (2014). “Auto-encoding Variational Bayes,” in 2nd International Conference on Learning Representations, ICLR 2014, Banff, AB, Canada, April 14-16, 2014. Editors Bengio Y. LeCun Y. Conference Track Proceedings.

[B18] Koren Y. Bell R. Volinsky C. (2009). Matrix Factorization Techniques for Recommender Systems. Computer 42, 30–37. 10.1109/MC.2009.263

[B19] Kumar A. Boehm M. Yang J. (2017). “Data Management in Machine Learning,” in Proc. ACM SIGMOD Int. Conf. Manag. Data Part, Chicago Illinois USA (Association for Computing Machinery), 1717–1722. F1277. 10.1145/3035918.3054775

[B20] Li S. C. Jiang B. Marlin B. M. (2019). “Misgan: Learning from Incomplete Data with Generative Adversarial Networks,” in 7th International Conference on Learning Representations, ICLR 2019, May 6-9, 2019 (New Orleans, LA, USA. (OpenReview.net).

[B21] Little R. J. A. Rubin D. B. (2002). Statistical Analysis with Missing Data. 2nd Edition. Hoboken: John Wiley & Sons.

[B22] Ma C. Tschiatschek S. Turner R. E. Hernández-Lobato J. M. Zhang C. (2020). “VAEM: a Deep Generative Model for Heterogeneous Mixed Type Data,” in Advances in Neural Information Processing Systems 33: Annual Conference on Neural Information Processing Systems 2020, December 6-12, 2020. Editors Larochelle H. Ranzato M. Hadsell R. Balcan M. Lin H. (NeurIPS, 2020). virtual.

[B23] Mazumder R. Hastie T. Tibshirani R. (2010). Spectral Regularization Algorithms for Learning Large Incomplete Matrices. J. Mach. Learn. Res. 11, 2287–2322. 21552465 PMC3087301

[B24] Miyato T. Kataoka T. Koyama M. Yoshida Y. (2018). “Spectral Normalization for Generative Adversarial Networks,” in 6th International Conference on Learning Representations, ICLR 2018, Vancouver, BC, Canada, April 30 - May 3, 2018. Conference Track Proceedings (OpenReview.net).

[B25] Nazábal A. Olmos P. M. Ghahramani Z. Valera I. (2020). Handling Incomplete Heterogeneous Data Using Vaes. Pattern Recognition 107, 107501. 10.1016/j.patcog.2020.107501

[B26] Pedregosa F. Varoquaux G. Gramfort A. Michel V. Thirion B. Grisel O. (2011). Scikit-learn: Machine Learning in Python. J. Mach. Learn. Res. 12, 2825–2830.

[B27] Poulos J. Valle R. (2018). Missing Data Imputation for Supervised Learning. Appl. Artif. Intelligence 32, 186–196. 10.1080/08839514.2018.1448143

[B28] Qiu Y. L. Zheng H. Gevaert O. (2020). Genomic Data Imputation with Variational Auto-Encoders. GigaScience 9, 1–12. 10.1093/gigascience/giaa082 PMC7407276 32761097

[B29] Rubin D. B. (1976). Inference and Missing Data. Biometrika 63, 581–592. 10.1093/biomet/63.3.581

[B30] Rubin D. B. (1987). Multiple Imputation for Nonresponse in Surveys. New York: Wiley.

[B31] Rukat T. Lange D. Schelter S. Biessmann F. (2020). “Towards Automated Data Quality Management for Machine Learning,” in ML Ops Work. Conf. Mach. Learn. Syst., 1–3.

[B32] Salimans T. Goodfellow I. J. Zaremba W. Cheung V. Radford A. Chen X. (2016). “Improved Techniques for Training gans,” in Advances in Neural Information Processing Systems 29: Annual Conference on Neural Information Processing Systems 2016, Barcelona, Spain, December 5-10, 2016. Editors Lee D. D. Sugiyama M. von Luxburg U. Guyon I. Garnett R., 2226–2234.

[B33] Schafer J. L. Graham J. W. (2002). Missing Data: Our View of the State of the Art. Psychol. Methods 7, 147–177. 10.1037/1082-989x.7.2.147 12090408

[B34] Schelter S. Biessmann F. Januschowski T. Salinas D. Seufert S. Szarvas G. (2018a). On Challenges in Machine Learning Model Management. IEEE Data Eng. Bull. 41 (4), 5–15. http://sites.computer.org/debull/A18dec/p5.pdf

[B35] Schelter S. Böse J.-H. Kirschnick J. Klein T. Seufert S. (2017). Automatically Tracking Metadata and Provenance of Machine Learning Experiments. Mach. Learn. Syst. Work. NIPS, 1–8.

[B36] Schelter S. Lange D. Schmidt P. Celikel M. Biessmann F. Grafberger A. (2018b). Automating Large-Scale Data Quality Verification. Proc. VLDB Endow. 11, 1781–1794. 10.14778/3229863.3229867

[B37] Schelter S. Rukat T. Biessmann F. (2021). “JENGA - A Framework to Study the Impact of Data Errors on the Predictions of Machine Learning Models,” in Proceedings of the 24th International Conference on Extending Database Technology, EDBT 2021, Nicosia, Cyprus, March 23 - 26, 2021. Editors Velegrakis Y. Zeinalipour-Yazti D. Chrysanthis P. K. Guerra F. (OpenProceedings.org), 529–534. 10.5441/002/edbt.2021.63

[B38] Schelter S. Rukat T. Biessmann F. (2020). “Learning to Validate the Predictions of Black Box Classifiers on Unseen Data,” in Proc. 2020 ACM SIGMOD Int. Conf. Manag. Data (New York, NY, USA: ACM)), 1289–1299. 10.1145/3318464.3380604

[B39] Sculley D. Holt G. Golovin D. Davydov E. Phillips T. Ebner D. (2015). Hidden Technical Debt in Machine Learning Systems. Adv. Neural Inf. Process. Syst. 2, 2503–2511. 2015-Janua.

[B40] Sculley D. Snoek J. Wiltschko A. B. Rahimi A. (2018). “Winner’s Curse? on Pace, Progress, and Empirical Rigor,” in ICLR Workshops.

[B41] Shang C. Palmer A. Sun J. Chen K.-S. Lu J. Bi J. (20172017). “VIGAN: Missing View Imputation with Generative Adversarial Networks,” in 2017 IEEE International Conference on Big Data, BigData 2017, Boston, MA, USA. Editors Nie J. Obradovic Z. Suzumura T. Ghosh R. Nambiar R. Wang C. Zang H. Baeza-Yates R. Hu X. Kepner J. Cuzzocrea A. Tang J. Toyoda M. (IEEE Computer Society), 766–775. 10.1109/BigData.2017.8257992 PMC5813842 29457155

[B42] Stekhoven D. J. Bühlmann P. (2012). MissForest--non-parametric Missing Value Imputation for Mixed-type Data. Bioinformatics 28, 112–118. 10.1093/bioinformatics/btr597 22039212

[B43] Stoyanovich J. Howe B. Jagadish H. V. (2020). Responsible Data Management. Proc. VLDB Endow. 13, 3474–3488. 10.14778/3415478.3415570

[B44] Troyanskaya O. Cantor M. Sherlock G. Brown P. Hastie T. Tibshirani R. (2001). Missing Value Estimation Methods for DNA Microarrays. Bioinformatics 17, 520–525. 10.1093/bioinformatics/17.6.520 11395428

[B45] van Buuren S. (2018). Flexible Imputation of Missing Data. 2nd ed. Boca Raton, FL: CRC Press.

[B46] Vanschoren J. van Rijn J. N. Bischl B. Torgo L. (2014). OpenML. SIGKDD Explor. Newsl. 15, 49–60. 10.1145/2641190.2641198

[B47] Wang Z. Akande O. Poulos J. Li F. (2021). Are Deep Learning Models superior for Missing Data Imputation in Large Surveys? Evidence from an Empirical Comparison. CoRR abs/2103.09316.

[B48] Woznica K. Biecek P. (2020). Does Imputation Matter? Benchmark for Predictive Models. CoRR abs/2007.02837.

[B49] Yang K. Huang B. Schelter S. (2020). Fairness-Aware Instrumentation of Preprocessing Pipelines for Machine Learning. 10.1145/3398730.3399194

[B50] Yin P. Neubig G. Yih W.-t. Riedel S. (2020). “Tabert: Pretraining for Joint Understanding of Textual and Tabular Data,” in Proceedings of the 58th Annual Meeting of the Association for Computational Linguistics, ACL 2020, July 5-10, 2020. Editors Jurafsky D. Chai J. Schluter N. Tetreault J. R. (Association for Computational Linguistics), 8413–8426. Online. 10.18653/v1/2020.acl-main.745

[B51] Yoon J. Jordon J. van der Schaar M. (2018). “GAIN: Missing Data Imputation Using Generative Adversarial Nets,” in Proceedings of the 35th International Conference on Machine Learning, ICML 2018, Stockholmsmässan, Stockholm, Sweden, July 10-15, 2018. Editors Dy J. G. Krause A. (vol. 80 of Proceedings of Machine Learning Research), 5675–5684.

[B52] Zhang H. Xie P. Xing E. P. (2018). Missing Value Imputation Based on Deep Generative Models. CoRR abs/1808.01684.

[B53] Ziemann M. Eren Y. El-Osta A. (2016). Gene Name Errors Are Widespread in the Scientific Literature. Genome Biol. 17. 10.1186/s13059-016-1044-7 PMC4994289 27552985

