# Supplementary material for: A Benchmark for Data Imputation Methods
Source: Front Big Data. 2021 Jul 8;4:693674. doi: 10.3389/fdata.2021.693674 (PMC8297389; doi:10.3389/fdata.2021.693674)
Supplement: Supplementary file 1 [file DataSheet1.PDF]

Supplementary Material

1 DATASETS

Tables S1 to S3 present all data sets we use for our benchmark suit.

OpenML ID	Name	To-be-imputed Column	Obs.	Features	
				Num.	Cat.
42545	stock_fardamento02	Material	6277	6	1
42675	auml_eml_1_d	F4_plsa_topic_10	4585	11	0
198	delta_elevators	curRoll	9517	7	0
23515	sulfur	a3	10081	7	0
189	kin8nm	theta8	8192	9	0
287	wine_quality	sulphates	6497	12	0
42636	Long	x18	4477	20	0
42688	Brazilian_houses	area	10692	9	4
42183	dataset_sales	day	10738	15	0
1199	BNG(echoMonths)	wall_index	17496	7	3
197	cpu_act	pgin	8192	22	0
218	house_8L	P3	22784	9	0
42712	Bike_Sharing_Demand	humidity	17379	9	4
1193	BNG(lowbwt)	UI	31104	3	7
216	elevators	climbRate	16599	19	0
215	2dplanes	x6	40768	11	0
23395	COMET_MC_SAMPLE	wire_id	89640	6	0
42225	diamonds	carat	53940	7	3
1200	BNG(stock)	company1	59049	10	0
1213	BNG(mv)	x5	78732	8	3
42669	auml_url_2	mld.ps_res	95911	13	0

Table S1. Overview of all data sets we use associated with a regression downstream task. *To-be-imputed Column* presents the data set's column we used to evaluate the imputation methods, *Obs.* the number of observations, *Num.* the number numerical columns, and *Cat.* the number of categorical columns.

OpenML ID	Name	To-be-imputed Column	Obs.	Features	
				Num.	Cat.
737	space_ga	INCOME	3107	6	1
871	pollen	RIDGE	3848	5	1
40983	wilt	Mean_G	4839	5	1
728	analcatdata_supreme	Lower_court_disagreement	4052	7	1
1489	phoneme	V1	5404	5	1
803	delta_ailerons	RollRate	7129	5	1
923	visualizing_soil	isns	8641	3	2
725	bank8FM	a2pop	8192	8	1
42192	compas-two-years	age	5278	7	7
1558	bank-marketing	V7	4521	7	10
310	mammography	attr2	11183	6	1
1046	mozilla4	end	15545	5	1
847	wind	year	6574	14	1
40701	churn	total_day_minutes	5000	16	5
41146	sylvine	V4	5124	20	1
1496	ringnorm	V4	7400	20	1
1507	twonorm	V5	7400	20	1
823	houses	total_rooms	20640	8	1
42493	airlines	Length	26969	2	6
1471	eeg-eye-state	V9	14980	14	1
1120	MagicTelescope	fAlpha:	19020	11	1
4135	Amazon_employee_access	MGR_ID	32769	0	10
137	BNG(tic-tac-toe)	top-middle-square	39366	0	10
251	BNG(breast-w)	Cell_Shape_Uniformity	39366	9	1
1220	Click_prediction_small	keyword_id	39948	9	1
151	electricity	nswprice	45312	7	2
901	fried	X2	40768	10	1
881	mv	x3	40768	7	4
40922	Run_or_walk_information	gyro_y	88588	6	1
42477	default-of-credit-card-clients	x1	30000	23	1
23517	numerai28.6	attribute_10	96320	21	1

Table S2. Overview of all data sets we use associated with a binary classification downstream task. *To-be-imputed Column* presents the data set's column we used to evaluate the imputation methods, *Obs.* the number of observations, *Num.* the number numerical columns, and *Cat.* the number of categorical columns.

OpenML ID	Name	To-be-imputed Column	Obs.	Features	
				Num.	Cat.
1526	wall-robot-navigation	V3	5456	4	1
183	abalone	Length	4177	7	2
40498	wine-quality-white	V11	4898	11	1
30	page-blocks	eccen	5473	10	1
40677	led24	attribute#3	3200	0	25
1459	artificial-characters	V7	10218	7	1
40497	thyroid-ann	V13	3772	21	1
4552	BachChoralHarmony	V2	5665	2	15
26	nursery	parents	12960	0	9
375	JapaneseVowels	coefficient3	9961	14	1
32	pendigits	input4	10992	16	1
1481	kr-vs-k	V2	28056	3	4
184	kropt	black_king_col	28056	0	7
41027	jungle_chess_2pcs_raw_endgame_(...)	black_piece0_strength	44819	6	1
6	letter	x-box	20000	16	1
41671	microaggregation2	a9	20000	20	1
40685	shuttle	A2	58000	9	1

Table S3. Overview of all data sets we use associated with a multi-class classification downstream task. *To-be-imputed Column* presents the data set's column we used to evaluate the imputation methods, *Obs.* the number of observations, *Num.* the number numerical columns, and *Cat.* the number of categorical columns.
